# Dietary Intake of the Urban Black Population of Cape Town: The Cardiovascular Risk in Black South Africans (CRIBSA) Study

**DOI:** 10.3390/nu8050285

**Published:** 2016-05-13

**Authors:** Nelia P. Steyn, Nasreen Jaffer, Johanna Nel, Naomi Levitt, Krisela Steyn, Carl Lombard, Nasheeta Peer

**Affiliations:** 1 Division of Nutrition, Department of Human Biology, University of Cape Town, Cape Town 7925, South Africa; nasreen.jaffer@uct.ac.za; 2 Department of Logistics, University of Stellenbosch, Stellenbosch 7600, South Africa; jhnel@sun.ac.za; 3 Division of Endocrinology and Diabetes, Department of Medicine, University of Cape Town, Cape Town 7925, South Africa; naomi.Levitt@uct.ac.za (N.L.); Krisella.steyn@uct.ac.za (K.S.); 4 Biostatistics Unit, South African Medical Research Council, Cape Town 7505, South Africa; Carl.Lombard@mrc.ac.za; 5 Non-communicable Diseases Research Unit, South African Medical Research Council, Durban 4001, South Africa; nasheeta.peer@mrc.ac.za

**Keywords:** South Africa, black, urban, dietary intake, energy, fats, carbohydrates, nutrition transition

## Abstract

Introduction: To determine dietary intake of 19 to 64 years old urban Africans in Cape Town in 2009 and examine the changes between 1990 and 2009. Methods: A representative cross-sectional sample (*n* = 544), stratified by gender and age was randomly selected in 2009 from the same areas sampled in 1990. Socio-demographic data and a 24-h dietary recall were obtained by trained field workers. The associations of dietary data with an asset index and degree of urbanization were assessed. Results: Fat intakes were higher in 19–44-year-old men (32% energy (E)) and women (33.4%E) in 2009 compared with 1990 (men: 25.9%E, women: 27.0%E) while carbohydrate intakes were lower in 2009 (men 53.2%E, women: 55.5%E) than in 1990 (men: 61.3%E; women: 62%E) while sugar intake increased significantly (*p* < 0.01) in women. There were significant positive correlations between urbanization and total fat (*p* = 0.016), saturated fat (*p* = 0.001), monounsaturated fat (*p* = 0.002) and fat as a %E intake (*p* = 0.046). Urbanization was inversely associated with intake of carbohydrate %E (*p* < 0.001). Overall micronutrient intakes improved significantly compared with 1990. It should also be noted that energy and macronutrient intakes were all significant in a linear regression model using mean adequacy ratio (MAR) as a measure of dietary quality in 2009, as was duration of urbanization. Discussion: The higher fat and lower carbohydrate %E intakes in this population demonstrate a transition to a more urbanized diet over last two decades. These dietary changes reflect the nutrition transitions that typically occur as a longer time is spent in urban centers.

## 1. Introduction

Diet and nutrition factors play a key role in the causation of non-communicable diseases (NCDs), diabetes, and cancers, by influencing the biological variables that mediate the risk for these conditions. A diet that is energy-dense, high in total fat and saturated fat, high in sugar and low in fiber and micronutrients frequently leads to obesity, which, in turn, is a major risk factor for the development of NCDs, including type 2 diabetes, certain cancers, and heart disease [1].

Over the past century, with rising industrialization, urbanization, and mechanization evident in most countries worldwide, diet and nutritional status have undergone major changes [1,2]. The changing dietary patterns are a product of the modern trade systems and the effect of the global food industry on food-supply chains [3]. Consequently, there has been a shift away from traditional diets toward the higher fat and higher refined carbohydrate Western diet [4], with dietary fat intake increasing steadily over the last four decades [5].

The pace of dietary change is occurring at varying degrees in different regions of the world [2]. While the proportion of fat intake has increased with growing country level income, unhealthy diets have been rising quickly in lower-resource settings, with particularly rapid increases in fat intake in lower-middle-income countries since the 1980s [5]. Growing incomes in developing regions have led to increases in the accessibility and intake of energy-dense high-fat diets, especially among the poor [1]. Intake of unhealthy diets that are richer in high-fat, high-energy foods contributes directly to increased energy imbalances, and the rapidly rising global levels of obesity and subsequent cardiometabolic diseases [6].

Additionally, inadequate micronutrient intake is common in developing regions of the globe and contributes to micronutrient deficiencies in both adults and children. Common among these are iron, zinc, calcium, folate, and vitamins A and D [7]. Multiple deficiencies can add to the compromise of nutritional health and further add to the burden of diseases.

In South Africa, the uptake of such unhealthy diets are evident by their contribution to the rising burden of NCDs and their risk factors, as reported in the national South African Health and Nutrition Examination Survey (SANHANES) [8].

It is important to monitor dietary patterns in a community so as to identify problem areas, which may be rectified through community education or government policies. Strategies that encourage optimal healthy eating patterns to reduce the risk for NCDs can be introduced, as well as foods rich in the necessary micronutrients, can be made cheaper and more easily accessible. However, detailed dietary data at a population level were last obtained in the black population of Cape Town in 1990, in the Black Risk Factor (BRISK) study [9,10].

In 2009, the Cardiovascular Risk in Black South Africans (CRIBSA) study was undertaken in the same areas where the 1990 BRISK study was done in order to determine, not only the prevalence of NCDs, but also the occurrence of lifestyle risk factors associated with them. It was found that the prevalence of overweight and obesity had increased in women from 72.7% in 1990 to 82.6% in 2009, while it decreased in men from 37.3% to 27.7% [11]. Age standardized diabetes prevalence increased by 53% in 2009 compared with 1990, and impaired glucose tolerance by 67% [12]. The prevalence of hypertension in 25 to 64 years olds was significantly higher in 2009 (35.6%) compared with 21.6% in 1990 [13]. Furthermore, the prevalence of raised low density lipoprotein cholesterol (LDL-C) and reduced high density lipoprotein cholesterol (HDL-C) prevalence increased significantly between 1990 and 2009 [14].

The present study focused on the dietary intake of the black population in CRIBSA 2009 and examined dietary changes between 1990 [9,10] and 2009, nearly two decades later. The researchers hypothesized that the diet would become more atherogenic the longer one lived in the city.

## 2. Materials and Methods

### 2.1. Sample

The sample size was determined according to an estimated diabetes prevalence of 8% in this population. The sampling frame was based on the 1990 Study [9,10] to ensure comparability, and included participants living in the townships of Langa, Gugulethu, Khayelitsha, Crossroads and Nyanga. There was proportionate sampling of these areas according to their population densities.

Aerial maps of the selected townships were used to randomly select the households. Individuals from these households were selected on a quota for age and gender categorization; the rationale being that the risk factors and disease prevalence differ among age groups and gender.

The following adults were excluded: Pregnant and lactating women; those who were bedridden; those unable to give consent; those on tuberculosis treatment; those on antiretroviral therapy; cancer patients having received treatment within the last year; and individuals residing in Cape Town for less than three months.

### 2.2. Dietary Intake

Each participant completed a 24-h recall conducted by fieldworkers who had been trained by a dietician with many years of research experience. The participant was required to recall all foods and drinks consumed during the past 24 h using the multiple pass method [15]. The 24-h recall dietary method was used for comparability with the 1990 study, which used the same method. Visual life-size photographs and sketches of foods and measures (such as cups, glasses) were used to identify portion sizes [16]. At the end of the interview, the interviewer summarized and probed for forgotten foods. The data was coded and the South African food composition tables [17] were used to calculate the dietary intake of every person and adjusted for fortification values. In order to determine dietary quality the values of macro- and micronutrients were compared with the dietary reference intakes (DRIs) including the recommended dietary allowances (RDA), the estimated average intakes (EARs), adequate intakes (AIs) and the acceptable macronutrient distribution ranges (AMDRs) [18]. The latter provided information on acceptable dietary ranges for carbohydrate, fat, and protein, as a percentage of energy intake. Dietary quality was also assessed by determining the nutrient adequacy ratio (NAR) of each micronutrient [19]. This was based on the mean % of the EAR value of the nutrient. For example, if a nutrient had a mean NAR of 75% then it met the EAR value by 75%. A 100% met the EAR value completely. The mean adequacy ratio (MAR) of the diet comprised the sum of the NAR values, truncated to 100%, divided by 11. Unfortunately, we were unable to access the MAR of the 1990 data since we did not have access to the original data.

### 2.3. Data Analyses

All dietary questionnaires were checked, coded, computerized and cleaned by an experienced dietician. The dietary data was then re-analyzed by using Goldberg’s method to remove the under-reporters [20]. This was done for two reasons: Firstly, the 24-h recall is known to underestimate dietary intake; and, secondly, energy intakes of the study participants were found to be very low, frequently lying well below resting energy expenditure (REE). The Goldberg equation includes dividing energy intake (EI) by estimated energy requirement (EER). Under-reporters are those regarded as falling below 0.8. To ensure comparability of the current study with the 1990 study, which was conducted in 15–64-year-old participants [9,10], the common age categories of 19–64 years were used for this analysis. *t* tests were used to determine significant differences between mean dietary values in 1990 compared with 2009.

The associations of different nutrient intakes with asset index, a proxy for wealth, and the duration of urbanization were assessed. A principal component analysis of the pooled data, based on the assets that defined wealth, was used to develop an asset index [21]. This was categorized into tertiles with relative wealth classified as poorest, poor and least poor. In lieu of the absence of an internationally agreed definition on what constitutes an urban environment, the proportion of life spent in the city, used in previous studies, defined the degree of urbanization [22]. Length of urban residence was recorded by summing the duration lived in a city, from birth until the date of data collection. We further computed a linear regression model using MAR as the dependent variable and added age, gender, urbanization, asset index and other variables to the model. Correlations between the asset index and urbanization duration were done with energy and nutrient intakes using Pearson’s correlations.

The University of Cape Town’s Research and Ethics Committee approved the study (REC REF: 224/2006). All participants signed informed consent.

## 3. Results

Dietary data was collected on 1097 participants in the CRIBSA study. Of these, 1009 were between 19 and 64 years of age. After removal of the under-reporters 544 participants (214 men and 330 women) aged between 19 and 64 years were included in the final sample for analysis. Participant distribution by area was as follows: Khayalitsha 42.4%, Langa 31.4%, Gugulethu 15.3% and less than 10% from Nyanga and Crossroads. Twelve percent of participants had lived in the city less than 20% of their lives, 44.5% between 20% and 69% of their lives, and 43.3% for 70% to 100% of their lives. By relative wealth, the poorest, poor and least poor participants comprised 30%, 54.8% and 14.8%, respectively, of the study sample.

Energy and macronutrient intakes are shown in Table 1. Mean energy intakes were on the low side in men while they appeared to be more moderate in women when compared with recommended values. Mean protein intakes were above the RDA values with nearly half coming from plant sources. Mean protein intakes and protein %E were significantly lower (*p* < 0.01) in men and women in 2009 compared with 1990.

Mean fat intake in 2009 (M = 70.3 g; W = 66.4 g) in the younger (19–44 years) group was significantly higher than in 1990 (M = 60 g; W = 49 g; *p* < 0.01). A similar scenario was found for fat %E. Saturated fat intake was lower in 2009 in men than in 1990. This difference was significant in the older (45–64 years) group (*p* < 0.01). In women, saturated fat intake was significantly higher in 2009 compared with 1990 (*p* < 0.0001) in both age groups. Fat %E, while within the recommended range, was towards the upper limit of the fat AMDR of 20%–35% in women and younger men. Saturated fat %E was above the recommended value of <7%E. Polyunsaturated fat intake and polyunsaturated fat %E were significantly higher in men and women in 2009 (*p* < 0.0001) compared with 1990. Similar results were found for the diet polyunsaturated/saturated ratio. Cholesterol intake, at >300 mg per day, was significantly higher in younger men and women in 2009 compared with 1990 (*p* < 0.001).

Mean carbohydrate intakes (which included added sugar) were nearly double the EAR values and were significantly lower in young men in 2009 (247.8 g *vs*. 282 g; *p* < 0.001) compared with 1990, while in women they were significantly higher in 2009 in younger (232.4 *vs*. 214 *p* < 0.01) and older women (221.4 g *vs*. 198 g; *p* < 0.01). Mean carbohydrate %E falls within the AMDR of 45%–65% and while not significant were lower in 2009 than in 1990. However added sugar is greater than the WHO [1] recommendation of less than 10%, with the exception of the younger men. Added sugar intake increased significantly in women in 2009 (*p* < 0.01), while it remained similar in men in the two studies. Mean fiber intakes were considerably lower than the AI of 25 g, in both studies.

Mean calcium intakes were very low in both studies lying below the AI of 1000 mg (Table 2). Values for men in 1990 were significantly higher than for men in 2009. Mean iron intakes were above the EAR in both studies. Vitamin A values were above the EARs in all groups with the exception of older men in 1990. Thiamin, riboflavin, niacin, vitamin B6, folate and vitamin C fell below the EARs in women in the 1990 study. In men in 1990, vitamin B6, folate and vitamin C were less than the EARs. Significant differences in mean intakes were noted between the two studies, which were generally higher in the 2009 study. This is notable for iron, folate, vitamin B6, niacin, thiamin, riboflavin and vitamin A, where there were significantly higher intakes compared with 1990. Calcium intakes remained low and zinc intakes were lower than the 1990 study.

Table 3 presents the NARs for the micronutrients. Generally the NAR values are highest in men in the 2009 study, followed by women in the 2009 study and lowest in women in the 1990 study. The mean NAR for vitamin A was above 100% in all groups with the exception of older men in 1990. Other nutrients that had NARs less than 100% were vitamin C, calcium and folate. In women in the 1990 study, calcium, vitamin B6, vitamin C, folate, riboflavin and thiamin were less than 100%.

The numbers of food portions (based on diabetic exchanges) eaten per day from five groups are shown in Table 4. The highest number of portions consumed in the 2009 study was from the cereal group with men and women having 9.45 and 8.73 mean portions per day, respectively. Cereals were followed by the fat group, with 3.30 and 4.20 mean portions per day for men and women, respectively. Vegetables and fruit are next with 2.78 and 2.90 portions respectively, followed by the meat group and lastly the dairy group with both men and women consuming less than one portion a day. For both men and women the portions in the 2009 study were significantly lower than those of the 1990 study for milk products, meat group, legumes, cereals (men only), saturated fats and brick margarine. However, the 2009 study had significantly higher intakes of eggs, vitamin C rich fruits and vegetables, cereals (women only), and polyunsaturated sources.

Correlations of nutrients with duration of urbanization (and the asset index score) were done to test for significant associations) (Table 5). Total energy intake and carbohydrate intake were not associated with duration of urbanization or with the asset index. However, total protein and fat intake were significantly associated with both. Significant positive correlations were found between most sub-groups of protein and fat with urbanization and the asset index except for polyunsaturated fat and cholesterol. The polyunsaturated fat/saturated fat ratio, carbohydrate %E, added sugar and sugar %E were inversely correlated with urbanization as well as with the asset index, except for added sugar. Sodium, zinc, thiamine, niacin and vitamin B6 were significantly associated with urbanization while calcium, zinc, thiamine and niacin were significantly associated with the asset index.

A linear regression model is presented in Table 6 with MAR as the dependent variable. Kilojoules and total fat were found to be highly correlated with total protein, and if kilojoules and total fat are included in the regression model, it results in multicolinearity, which lead to insignificant relationships between MAR and kilojoules and total fat, respectively. It is more useful to delete them from the model. Similarly, urbanization duration and asset index are highly correlated, and only one of the two variables need to be included in the model. For every BMI unit increase the mean MAR will increase by 0.23%. For every gram of protein consumed the mean MAR will increase by 0.26%. While for every gram of protein the MAR will increase by 0.03%. However, for every one-gram increase in the intake of added sugar, the mean MAR will decrease by 0.04%. The average MAR is 2.86% higher for females than males. These relationships are true if all other variables remain constant. Energy and macronutrients were all significant in the regression model, as was duration of urbanization.

## 4. Discussion

The findings pertaining to dietary intake in the CRIBSA study are in keeping with the nutritional transition occurring in urban centers in South Africa. Unlike rural areas where commonly, traditional diets that are low in fats (<25% of energy intake) and sugar, and high in carbohydrates are consumed, in urban centers there is a shift with the adoption of more Western diets. These are higher in fat consumption (>25% of energy intake) and lower in carbohydrates [25] as mirrored in this study.

Indeed, this is further illustrated by the significant positive correlations between greater urbanization duration and total fat, saturated fat, monounsaturated fat, and fat as a percent of energy intake. A further reinforcement of this transition is the differences observed in fat intake between 1990 [10] and 2009. This rise is due to an increase in polyunsaturated fats since saturated fat intake remained similar in the two studies, although rising significantly in the younger women.

Additionally, the significant inverse correlation of carbohydrate (which includes added sugar) as a percent of energy intake with urbanization duration reinforces the shift to a Western diet in urban centers. Additionally, the proportion of carbohydrate intake was significantly lower in men in 2009 compared with 1990 reinforcing the nutritional transition. These higher proportions of fat and lower proportions of carbohydrate intake, highlight that over two decades the dietary intake in this population has transitioned to a more urbanized and Western diet [25].

In addition to urbanization, socio-economic advancements are also known to be associated with westernization of lifestyles and increased uptake of energy-dense foods high in fats [26]. These foods are likely to be more affordable to individuals with higher socioeconomic status (the affluent and better educated) but may still be out of the price range of lower socio-economic groups, particularly in developing regions [27]. This accords with these study findings that showed a significant association between wealth and fat intake.

The overall energy intake of this urban population was within the range of other South African urban reports. It was greater than those of urban participants in a study in Free State Province [28] (M = 7078 kJ; W = 6621 kJ), one of few recent studies in adult blacks, but lower than those of participants in urban North West Province (PURE study) [29] (M = 10,054 kJ; W = 9008 kJ).

Notably, the energy intake in men was similar in 1990 and 2009, which may account for the same obesity prevalence of 9.5% in the two studies [11]. In contrast, energy intake in women increased from 1990 [10] to 2009 as did obesity levels. However, energy intake was within the normal range and does not reflect the extremely high prevalence of obesity among women in this population (1990: 41.7%; 2009: 61.5%). It should also be noted that energy and macronutrient intakes were all significant in the linear regression model using MAR in 2009, as was duration of urbanization.

Mean protein intake in this study was lower than that reported in the Free State [28] (M = 76 g; W = 69 g), the North West [29] (M = 74 g; W = 64 g), and in the 1990 study [10] (M = 77 g; W = 56 g). This may be due to socio-economic reasons since protein-rich foods are generally more costly, particularly animal protein sources and dairy products.

In the 1990 study numerous mean micronutrient values were lower than the DRI values [9,10]. Low micronutrient levels in men included calcium, vitamin A, vitamin B6, folate and vitamin C. In women deficient nutrients included calcium, thiamin, riboflavin, niacin, vitamin B6, folate, and vitamin C. However, most of these improved substantially as evinced by the higher NAR values for iron, zinc, vitamin A, folate, vitamin B6, niacin, thiamin and riboflavin in the 2009 study. This is most probably the result of mandatory fortification of maize meal and wheat flour, which was introduced in 2004. Calcium and zinc intake however remain below the recommended values. Since both studies were undertaken in the city of Cape Town, it is not possible to make comparisons with the rural areas; however, the national food consumption study [30] in South Africa in 1999 did find that micronutrient deficiencies were more prevalent in rural areas.

The lower than recommended intake of fruits and vegetables in this study is of concern because adequate amounts are necessary to lower the risk of IHD, stroke and high blood pressure, as well as stomach and colorectal cancers [5,31,32]. Fruit and vegetables contribute to cardio-metabolic health through numerous phyto-nutrients, potassium and fiber [31,32].

In addition, the low meat group consumption in women could lead to possible low iron and vitamin B12 intakes. The very low consumption of dairy products explains the low mean calcium and riboflavin values in both studies. The portions of food groups consumed are also a reflection of urbanization of diet. As with the 1990 study, the lowest intakes are in the dairy group [9]. The 1990 study found that with increased time spent in the city dairy consumption decreased by 33% and cereal intake by 26% while fruit and vegetable intake increased by 19%, non-basic foods by 17%, fats by 8%, and meat by 14% [9].

The 1990 study determined that overall the dietary pattern of the urban black population generally met the prudent dietary recommendations of the American Heart Association (AHA) [33] namely that fat intake should be less than 30% of energy intake, saturated fats less than 10% energy intake and sugar less than 10% energy intake. To a large degree, the 1990 population was considered to be in a transition phase regarding their dietary intake since many participants had only lived in the city for a short period of time.

### Limitations

Limitations of the study include the use of a single 24 h recall, which is known to under-estimate dietary intakes [34]. Nonetheless, this method was used to ensure comparability with the 1990 study, which used only one 24 h recall. Another limitation was the fact that we did not have access to the original data of the 1990 data and had to rely on published data. Removal of under-reporters using the Goldberg method [20] could also be regarded as a limitation since the sample was nearly halved, which means the findings are not generalizable. This limitation was particularly noticeable when interpreting the mean energy intakes of the women. These values are within the normal to lower range and do not actually reflect the high prevalence of obesity found in women in this study. However, it has been shown that obese women do tend to under-report their intake [35]. It is also likely that low physical activity levels may have contributed in this regard since the national survey [8] did find a low prevalence of physical activity in African women.

## 5. Conclusions

The nutrient intakes demonstrate that while certain changes have taken place between 1990 and 2009, the dietary pattern regarding foods eaten remains poor. The diet has become more urbanized and atherogenic with regard to distribution of fat and carbohydrate intake while the consumption of certain food groups have remained low, such as the poor consumption of dairy products and low intake of fruit and vegetables. On the other hand, low micronutrient intakes have increased over the two time periods as a result of mandatory fortification of staple foods in 2004. Interventions are urgently needed to combat the shift towards the diet continuing to become more atherogenic and to improve the consumption of priority food groups, such as dairy products and fruit and vegetables.

## Figures and Tables

**Table 1:** Energy and macronutrient intakes of 19–64-year-old black adults in the Cardiovascular Risk in Black South Africans (CRIBSA) study (2009) in Cape Town and comparison with the Black Risk Factor (BRISK) 1990 study [10].

Age Groups	Men 2009		Men 1990		DRIs ^1^	Women 2009		Women 1990		DRIs ^1^
	19–44	45–64	19–44	45–64		19–44	45–64	19–44	45–64	
*N*	138	76	285	98		216	114	364	117	
Energy (kJ)	8557 ± 2971	7666 ± 2219	8500 ± 3700	9196 ± 3800	10,609 kJ ^2^	7619 ± 2271 **	7104 ± 1838 ***	6400 ± 2800	6400 ± 2000	7971 kJ ^3^
Protein (g)	64.5 ± 29.0 **	57.0 ± 27.4 **	77 ± 44.0	78 ± 51.0	RDA ^4^ = 56 g	52.8 ± 23.4	48.7 ± 18.0	56 ± 33.0	49 ± 21.0	RDA ^4^ = 46 g
Protein %E ^5^	13.7 ± 4.8 *	13.4 ± 5.1 **	15.1 ± 4.8	15.3 ± 5.4	10%E–35%E ^5^	12.4 ± 4.5 **	12.7 ± 4.9 *	14.5 ± 4.8	14.3 ± 3.3	10%E–35%E ^5^
Plant Protein (g)	26.9 ± 11.0 ***	25.6 ± 10.6 *	34 ± 21.0	31 ± 18.0		23.6 ± 8.9	22.9 ± 8.2	23 ± 14.0	22 ± 10.0	
Animal Protein (g)	35.5 ± 26.5 *	29.1 ± 25.8 **	42 ± 40.0	46 ± 49.0		26.4 ± 21.3 *	23.4 ± 17.7	33 ± 30.0	28 ± 19.0	
Fat (g)	70.3 ± 41.2 *	52.9 ± 35.0	60 ± 43.0	57 ± 43.0		66.4 ± 38.4 ***	59.9 ± 38.9 ***	49 ± 33.0	42 ± 25.0	
Fat %E	32.0 ± 12.1 ***	27.2 ± 14.0	25.9 ± 11.8	23.8 ± 11.7	20%E–35%E ^5^	33.4 ± 11.8 ***	32.6 ± 14.1 ***	27 ± 11.2	26.1 ± 9.6	20%E–35%E ^5^
Saturated fat (g)	18.6 ± 12.5	14.5 ± 10.3 *	20 ± 17.0	21 ± 18.0		17.3 ± 11.5 *	17.3 ± 16.2 *	16 ± 12.0	15 ± 10.0	
Saturated fat %E ^5^	8.5 ± 4.3	7.5 ± 4.5	8.6 ± 4.4	8.8 ± 4.8	<7%E ^5^	8.6 ± 3.8 *	9.6 ± 7.9	8.8 ± 4.3	9.2 ± 3.9	<7%E ^5^
MUFA ^6^ (g)	22.6 ± 14.6	16.9 ± 12.8	21 ± 17.0	20 ± 18.0		21.7 ± 14.1 **	17.7 ± 11.7	17 ± 14.0	15 ± 11.0	
MUFA %E	10.2 ± 4.8 **	8.6 ± 5.5	9 ± 5.2	8.3 ± 5.0		10.8 ± 4.7 ***	9.8 ± 5.2	9.4 ± 5.0	9.3 ± 4.0	
PUFA ^7^ (g)	23.5 ± 19.8 ***	17.3 ± 16.4 **	13 ± 10.0	10 ± 9.0		22.6 ± 17.1 ***	20.6 ± 21.7 ***	11 ± 9.0	8 ± 5.0	
PUFA %E ^5^	10.7 ± 6.8 ***	8.8 ± 6.9 ***	5.7 ± 3.7	4.5 ± 3.4		11.5 ± 6.7 ***	10.8 ± 7.8 ***	6.3 ± 3.9	5.4 ± 3.0	
Diet P/S ratio	1.53 ± 1.1 ***	1.52 ± 1.3 ***	0.81 ± 0.67	0.73 ± 0.60		1.66 ± 1.13 ***	1.56 ± 1.33 ***	0.88 ± 0.75	0.77 ± 0.58	
Cholesterol (mg)	359.8 ± 346 **	258.5 ± 322	265 ± 288	260 ± 270	300 mg	285.9 ± 326 ***	216.3 ± 227	213 ± 226	174 ± 136	300 mg
Carbohydrate (g)	247.8 ± 97.9 **	237.5 ± 67.4	282 ± 128	266 ± 112	EAR ^8^ = 100	232.4 ± 66.4 *	221.4 ± 65.9 *	214 ± 95.0	198 ± 73.0	EAR ^8^ = 100 g
					RDA ^4^ = 130					RDA ^4^ = 130 g
Carbohydrate %E ^5^	53.2 ± 13.7	57.4 ± 14.1	61.3 ± 16.3	59.2 ± 16.6	45%E–65%E ^5^	55.5 ± 12.5	57.3 ± 15.0	62 ± 15.3	62.7 ± 12.2	45%E–65%E
Fiber (g)	18.9 ± 10.4	18.1 ± 10.4	21 ± 15.0	19 ± 13.0	AI ^9^ = 38 g	16.2 ± 8.5	16.8 ± 8.2 ***	16 ± 11.0	13 ± 6.0	AI ^9^ = 25 g
Added Sugar (g)	45.0 ± 42.8	49.4 ± 37.7	50 ± 44.0	51 ± 39.0		54.4 ± 40.8 *	47.0 ± 36.3 *	47 ± 34.0	38 ± 24.0	
Sugar %E ^5^	9.5 ± 8.3	12.1 ± 9.3	11.0 ± 9.6	11.4 ± 10.1	<10%E ^5^	13.3 ± 9.6	12.2 ± 8.9	13.6 ± 11.1	11.4 ± 6.6	<10%E ^5^

^1^ Dietary reference intakes; ^2^ Sedentary man of 1.75 m with BMI of 24.9 kg·m^2^; ^3^ sedentary woman of 1.60 m with BMI of 24.9 kg·m^2^; ^4^ RDA: Recommended dietary allowance; ^5^ Energy; ^6^ MUFA = monounsaturated fatty acids; ^7^ PUFA = polyunsaturated fatty acids; ^8^ EAR: Estimated average requirement; ^9^ AI: Adequate intake; * *p* < 0.05; ** *p* < 0.01; *** *p* < 0.0001 using the t-test between same age categories between the two studies.

**Table 2:** Mean micronutrient intake in 19–64-year-old men and women in 1990 [10] and 2009.

Age Groups	Men 2009		Men 1990			Women 2009		Women 1990		
	19–44	45–64	19–44	45–64	DRIs ^1^	19–44	45–64	19–44	45–64	DRIs ^1^
*N*	138	76	285	98		216	114	364	117	
Calcium (mg)	384 ± 307 ***	324 ± 245 **	526 ± 484	522 ± 409	AI ^2^ = 1000	312 ± 266	346 ± 275	337 ± 221	358 ± 208	AI = 1000
Iron (mg) ^5^	13.3 ± 5.5 ***	11.4 ± 4.8	10 ± 6	10 ± 6	EAR ^3^ = 8.1	11.3 ± 4.8 ***	10.9 ± 3.7 ***	7 ± 4	7 ± 4	EAR = 6.0
Zinc (mg) ^5^	9.6 ± 4.5	8.5 ± 3.9 *	10.6 ± 8.4	11.8 ± 13.4	EAR = 9.4	7.8 ± 3.4	7.4 ± 2.4	7.7 ± 6.2	6.8 ± 4.1	EAR = 6.8
Vitamin A (ug) ^5^	1133 ± 3667	1198 ± 4243 *	724 ± 2829	395 ± 691	EAR = 625	1066 ± 2411 **	785 ± 1332	558 ± 1141	577 ± 1075	EAR = 500
Thiamin (mg) ^5^	1.2 ± 0.5 *	1.0 ± 0.4	1.1 ± 0.6	1.0 ± 0.6	EAR = 1.0	1.0 ± 0.5 **	1.0 ± 0.4 ***	0.9 ± 0.5	0.8 ± 0.3	EAR = 0.9
Riboflavin (mg) ^5^	1.7 ± 2.9 *	1.1 ± 1.6	1.2 ± 1.3	1.1 ± 0.7	EAR = 1.1	1.5 ± 2.3 ***	1.1 ± 0.8 ***	0.8 ± 0.6	0.8 ± 0.5	EAR = 0.9
Niacin (mg) ^5^	18.1 ± 8.9 *	16.0 ± 9.4	16.1 ± 11.6	15.1 ± 8.9	EAR = 12	14.1 ± 7.4 **	13.2 ± 6.2 ***	12.2 ± 8	10 ± 5.7	EAR = 11
Vitamin B6 (mg) ^5^	1.5 ± 0.7 ***	1.3 ± 0.7 *	1.2 ± 0.8	0.8 ± 0.1	EAR = 1.1	1.0 ± 0.6 *	0.8 ± 0.4 **	0.9 ± 0.6	0.8 ± 0.4	EAR = 1.1
Folate (ug) ^5^	335 ± 191 ***	324 ± 201 ***	218 ± 175	182 ± 147	EAR = 320	289 ± 254 ***	302 ± 173 ***	155 ± 131	147 ± 79	EAR = 320
Vitamin B12 (ug)	9.8 ± 38.2	9.6 ± 43.0	6.9 ± 29.3	3.8 ± 5.0	EAR = 2.0	5.8 ± 21.1	4.0 ± 10.6	3.6 ± 8.7	3.5 ± 8.9	EAR = 2.0
Vitamin C (mg)	83.3 ± 177.5	61.4 ± 77.4	54 ± 151	27 ± 59	EAR = 75	63.2 ± 74.6 *	69.3 ± 76.3 **	42 ± 84	32 ± 49	EAR = 60
Vitamin D (ug)	5.5 ± 6.4	2.8 ± 4.1	na ^4^	na ^4^	AI = 5	4.4 ± 5.8	3.1 ± 4.3	na ^4^	na ^4^	AI = 5
Vitamin E (mg)	17.8 ± 17.2	13.4 ± 14.9	na ^4^	na ^4^	EAR = 12	17.0 ± 15.5	16.8 ± 19.7	na ^4^	na ^4^	EAR = 12

^1^ DRI: Dietary reference intake; ^2^ AI: Adequate intake; ^3^ EAR: Estimated average intake; ^4^ na: Not available; ^5^ nutrients added by mandatory fortification since 2003 [23]; * *p* < 0.05; ** *p* < 0.01; *** *p* < 0.0001 using the t-test between same age categories between the two studies.

**Table 3:** Mean nutrient adequacy ratios (NARs) of micronutrient intakes in 19–64-year-old men and women, in 2009 and 1990 [10].

Age Groups: Years	Men 2009		Men 1990		Women 2009		Women 1990	
	19–44	45–64	19–44	45–64	19–44	45–64	19–44	45–64
*N*	138	76	285	98	216	114	364	117
NAR Vitamin A (%)	182	187	116	63	213	156	112	115
NAR Vitamin B6 (%)	106	93	109	73	89	77	82	73
NAR Vitamin B12 (%)	488	478	345	190	289	198	180	175
NAR Vitamin C (%)	111	82	72	36	105	115	70	53
NAR Calcium (%)	38	32	53	52	31	35	34	36
NAR Folate (%)	105	319	68	57	90	297	48	46
NAR Iron (%)	165	139	123	123	187	181	117	117
NAR Niacin (%)	151	133	134	126	128	120	111	91
NAR Riboflavin (%)	158	101	109	100	165	116	89	89
NAR Thiamin (%)	120	100	110	100	113	114	100	89
NAR Zinc (%)	103	90	113	126	114	108	113	100
MAR ^1^	77	71	na ^2^	na ^2^	75	76	na ^2^	na ^2^

^1^ NAR: Nutrient adequacy ratio: % of nutrient of the EAR value with 100% equaling 100% EAR value; MAR: mean adequacy ratio; MAR ^1^ for 1990 data calculated from [10]; ^2^ na: not available.

**Table 4:** Portion sizes ^1^ consumed by the study participants in 1990 [9] and in 2009.

	Men 2009	Men 1990	Women 2009	Women 1990	% Consumers 2009	% Consumers 1990
	*N* = 215	*N* = 441	*N* = 329	*N* = 542		
**Milk group**	0.43 ± 0.77 ***	0.9 ± 1.4	0.35 ± 0.64 **	0.5 ± 0.8	40.3	58
Milk	0.40 ± 0.76 ***	0.9 ± 1.4	0.34 ± 0.63 ***	0.5 ± 0.8	39.3	58
Cheese	0.03 ± 0.17	0.02 ± 0.2	0.01 ± 0.12 *	0.03 ± 0.2	2.4	4
**Meat group**	2.12 ± 1.85 **	2.6 ± 2.3	1.68 ± 1.44 *	1.9 ± 1.6	82.7	88
Red meat	0.68 ± 1.02	0.8 ± 1.7	0.48 ± 0.81	0.5 ± 1.0	42.8	29
White meat	0.60 ± 0.92	0.7 ± 1.0	0.54 ± 0.73	0.6 ± 0.8	46.0	41
Eggs	0.73 ± 1.34 ***	0.4 ± 1.0	0.53 ± 1.08 ***	0.3 ± 0.8	24.4	16
Legumes	0.10 ± 0.49 ***	0.5 ± 1.2	0.13 ± 0.42 ***	0.3 ± 0.8	9.9	20
**Vegetables & fruit**	2.78 ± 2.58 ***	2.1 ± 2.5	2.90 ± 2.52 **	2.4 ± 2.6	82.9	71
Vitamin C rich	0.57 ± 1.09 ***	0.2 ± 0.6	0.68 ± 1.01 ***	0.2 ± 0.6	47.8	21
Carotene rich	0.24 ± 0.65	0.2 ± 0.5	0.34 ± 0.69 **	0.2 ± 0.6	27.9	18
Potato/sweet potato	1.08 ± 1.73	1.0 ± 1.6	0.97 ± 1.42	1.0 ± 1.5	48.7	45
Other veg/fruit	0.89 ± 1.72	0.7 ± 1.5	0.94 ± 1.64	1.1 ± 1.8	39.2	46
**Cereal group**	9.45 ± 4.78 **	10.8 ± 6.9	8.73 ± 3.31 ***	7.5 ± 4.8	100.0	97
Unrefined	2.10 ± 2.40	2.0 ± 3.7	1.59 ± 1.85 **	1.2 ± 2.1	55.0	37
Refined	7.34 ± 5.04 **	8.7 ± 7.0	7.14 ± 3.71 **	6.4 ± 4.9	97.2	92
**Fat group**	3.30 ± 5.14	3.4 ± 3.9	4.20 ± 5.97	3.1 ± 3.2	59.4	77
Saturated fat	0.10 ± 0.63 ***	0.8 ± 2.1	0.32 ± 1.50 ***	0.7 ± 1.7	5.5	26
Brick margarine	1.31 ± 2.75 ***	2.1 ± 3.2	1.38 ± 3.12 **	1.9 ± 2.5	34.4	56
Oil/tub margarine	1.89 ± 4.73 ***	0.5 ± 1.4	2.50 ± 5.35 ***	0.6 ± 1.3	28.1	29

**^1^** Portion sizes calculated according to diabetic exchange lists [24]. * *p* < 0.05; ** *p* < 0.01; *** *p* < 0.0001.

**Table 5:** Pearson correlations of nutrients with urbanization duration and the asset index.

Nutrient	Urbanization ^1^		Asset Index ^2^	
	*R*^2^	*p*-Value	*R*^2^	*p*-Value
Energy (kJ)	0.061	0.161	0.040	0.354
**Protein**				
Total protein	0.168	<0.0001 ***	0.145	0.0008 **
Animal protein	0.186	<0.0001 ***	0.166	0.0001 **
Vegetable protein	−0.036	0.412	−0.105	0.015 *
Protein, %E ^3^	0.169	<0.0001 ***	0.143	0.0009 **
Animal %E ^3^	0.166	0.0001 **	0.217	<0.0001 ***
**Fat**				
Total fat	0.104	0.016 *	0.129	0.003 **
Saturated fat	0.141	0.001 **	0.189	<0.0001 ***
MUFA ^4^	0.132	0.002 **	0.147	0.0006 **
PUFA ^5^	0.004	0.917	0.008	0.855
Fat %E ^3^	0.086	0.046 *	0.159	0.0002 **
Saturated fat %E ^3^	0.136	0.001 **	0.212	<0.0001 ***
Cholesterol	0.036	0.401	0.028	0.511
MUFA ^4^ %E ^3^	0.133	0.0021 **	0.176	<0.0001 ***
PUFA ^5^ %E ^3^	−0.055	0.204	−0.020	0.645
P/S ratio ^6^	−0.151	0.0005 **	−0.189	<0.0001 ***
**Carbohydrate**				
Total carbohydrate	−0.084	0.052	−0.082	0.060
Fiber	0.009	0.840	−0.016	0.706
Added sugar	−0.095	0.028 *	−0.059	0.175
Carbohydrate, %E ^3^	−0.175	<0.0001 ***	−0.188	<0.0001 ***
Added sugar, %E ^3^	−0.120	0.006 **	−0.096	0.027 *
**Micronutrients**				
Calcium	0.058	0.181	0.116	0.007 **
Iron	0.074	0.086	0.060	0.164
Sodium	0.104	0.016 *	0.078	0.072
Zinc	0.154	0.0003 **	0.137	0.002 **
Vitamin A	−0.024	0.583	−0.008	0.854
Thiamin	0.140	0.001 **	0.134	0.002 **
Riboflavin	0.055	0.205	0.078	0.073
Niacin	0.167	0.0001 **	0.154	0.0004 **
Vitamin B6	0.1246	0.004 **	0.078	0.072
Folate	−0.034	0.427	−0.068	0.118
Vitamin B12	−0.015	0.724	−0.011	0.798

* *p* < 0.05; ** *p* < 0.01; *** *p* < 0.0001; ^1^ Urbanization duration: Time in years spent in the city. ^2^ Asset index: A principal component analysis of the pooled data, based on the assets that defined wealth, was used to develop an asset index [20]. ^3^ E: Energy; ^4^ MUFA: Monounsaturated fatty acids; ^5^ PUFA: Polyunsaturated fatty acids; ^6^ P/S ratio: Polyunsaturated fat/saturated fat ratio.

**Table 6:** Linear regression with mean adequacy ratio (MAR) of the diet as dependent variable for data from the 2009 study.

Variable	Parameter Estimate	*p*-Value Testing for the Significance of the Relationship in the Regression Model with MAR	Pearson Correlation with MAR	*p*-Value of Correlation Coefficient
Intercept	41.42	<0.0001 ***	-	
Urbanization ^1^	0.03	0.0238 *	0.1254	0.0034 **
Asset index ^2^			0.1790	<0.0001 ***
Body mass index	0.23	0.0017 **	0.1251	0.0035 **
Age	−0.06	0.1394	−0.0885	0.0391 *
Total protein	0.26	<0.0001 ***	0.5779	<0.0001 ***
Total energy			0.4421	<0.0001 ***
Total Fat			0.3029	<0.0001 ***
Added sugar	−0.04	0.0038 **	−0.210	0.6251
Gender	2.86	0.0110 *	0.0207	0.6307
Adjusted *R*^2^ = 0.4330				

* *p* < 0.05; ** *p* < 0.01; *** *p* < 0.0001; ^1^ Urbanization duration: Time in years spent in the city; ^2^ Asset index: A principal component analysis of the pooled data, based on the assets that defined wealth, was used to develop an asset index [20].
